# Unlocking the feed supplement potentials of blue-green alga (spirulina) in broiler nutrition: a comprehensive review

**DOI:** 10.1007/s11250-025-04587-1

**Published:** 2025-08-13

**Authors:** I. P. Ogbuewu, C. A. Mbajiorgu

**Affiliations:** 1 Department of Animal Science and Technology, Federal University of Technology, P.M.B. 1526, Owerri, Imo State Nigeria; 2 Department of Agriculture and Animal Health, University of South Africa, Florida Science Campus, Private Bag X6, Florida, 1710 South Africa

**Keywords:** Chemical composition, Productive indices, Health markers, Meat quality, Production economics

## Abstract

Presently, spirulina, a cyanobacterium, is gaining attention as a safe feed supplement in livestock and poultry production due to its rich nutritional and medicinal benefits. Spirulina grows well in highly alkaline environments with a pH range of 9.5–11 such as pond, fresh and marine water. It is an excellent source of proteins, vitamins (ascorbic acid, tocopherol, and B-complex), minerals (iron and magnesium), and fatty acids (gamma linoleic acid, capric acid, palmitic acid, omega-3 and omega-6). It has a higher essential amino acid profiles than soybean meal, which usually is the main protein source in poultry diets. In addition, spirulina contains phytopigments (carotenoids and phycocyanins) and bioactive compounds (polyphenols and flavonoinds), which have a wide range of biological activities, including antimicrobial, hypolipidemic, hypocholesterolemic, antioxidant, and anti-inflammatory effects. There has been variable results on growth-promoting effects of spirulina in broilers. Knowledge and detailed understanding of spirulina’s nutritional composition, (i.e., proximate, amino acid, vitamins, minerals, and fatty acid), mechanisms of action, and its supplementation value in broilers are critical to fully maximize its use in broiler nutrition. However, while most of these findings are valuable, they are scattered throughout the literature, making it difficult to use them in evidence-based decision-making in the poultry industry. The present review therefore attempts to pool current published evidence on spirulina’s chemical composition, and the effect of its supplementation on production indices and health status of broilers to enhance the adoption of these findings in decision support system in the poultry industry, as well as to identify knowledge gaps and fashion out new research directions. The phytochemical composition and mechanisms by which spirulina enhances broiler performance will also be reviewed.

## Introduction

Poultry products contribute immensely to world food production by providing high-quality protein and reducing food insecurity gap, particularly in developing countries. Chicken meat makes up 90 to 93% of poultry meat (FAO 2025). However, most households in developing countries still regard poultry products as luxury due to the high cost of meat driven by the high cost of feed (Abdulrahman et al. 2022). Feed alone accounts for more than 70% of the overall cost of chicken production (Mallick et al. 2020). This calls for introduction of feed supplements such as enzymes, probiotics, prebiotics, organic acids, herbs, or algae to enhance chicken performance. Among these, spirulina has emerged as a promising feed supplement and is gaining recognition in modern feeding practice.

Spirulina is a commercial strain of cyanobacteria noted for its high nutritional benefits. It is also known as blue-green algae. Spirulina thrives well in highly alkaline environments (with a pH range of 9.5–11) such as pond water, fresh water, and marine water. Spirulina also prefers moderate to high temperatures to grow optimally. Global spirulina production is estimated at 56, 208 tons in 2018 (FAO 2019). The world spirulina market size projected to attained € 533 million in 2023 (FAO 2019). Furthermore, spirulina is a sustainable feed and food supplement source, as it requires less land, water, and energy to produce. Research has shown that spirulina is high in proteins, B vitamins (B1, B2, and B3), and minerals (iron and magnesium) (Abdel-Moneim et al. 2022a; USDA 2024). Furthermore, spirulina is a rich source of carotenoids, ascorbic acid (vitamin C), phenolic compounds, and phycobiliproteins which play a vital part in neutralizing free radicals and mitigating oxidative stress (Zainoddin et al. 2018). The high levels of essential nutrients and bioactive constituents in spirulina may account for its diverse biological properties such as antioxidant, antimicrobial, immunomodulatory, and anti-inflammatory effects (Abdel-Moneim et al. 2022a). Spirulina’s antioxidant and anti-inflammatory properties can help mitigate oxidative stress and inflammation caused by environmental and nutritional stressors, resulting in improved chicken welfare (Abdel-Moneim et al. 2020; Hassan et al. 2023).

There are growing studies on feed supplement values of spirulina on broiler performance with conflicting findings (Moustafa et al. 2021; Abdel-Moneim et al. 2022a; Chaudhary et al. 2023; Hassan et al. 2023), These discrepancies may be related to factors like diet composition, growth conditions of spirulina, chicken genetics, quantity of spirulina included in the feed, and climatic variables (Rahim et al. 2021). Although most of these studies suggest that introduction of low levels of spirulina to diets improves broiler performance (Attia et al. 2023; Alwaleed et al. 2021; Kolluri et al. 2022; Spinola et al. 2024a), however, most of these studies are scattered in the literature, thereby making it difficult to use this valuable information in decision-support systems in the poultry industry. Therefore, the present review attempts to pool recently published results on the chemical composition of spirulina, its probable mechanisms of action, as well as the supplementation value of spirulina on growth dynamics, gut microbiota composition, intestinal morphology, blood characteristics, antioxidative enzyme biomarkers, immune functions, internal organ weights, carcass traits, and meat quality of broilers as to use this valuable information in decision support system in the poultry industry.

## Methodology

PubMed, Embase, Google, Web of Science (WoS), Scopus, ScienceDirect, and Google Scholar bibliographic databases were searched between 1st December 2024 and 10th March 2025 for published studies on the topic. The reference section of identified studies was also reviewed for other relevant articles. The search terms used were *Spirulina platensis*, spirulina algae, *Arthrospira platensis*, blue-green algae, Arthrospira, chemical composition, proximate analysis, proximate composition, amino acids, fatty acids, minerals, vitamins, pigments, broilers, broiler chickens, growth performance, feed intake, feed conversion efficiency, feed conversion ratio, blood characteristics, hematology, blood chemistry, serum biochemical values, organ weight, carcass yield, intestinal histology, gut morphology, gut microbiota, villus, carcass characteristics, meat quality, antioxidative status, and lipid profiles. The search terms were blended with Boolean logic (AND/OR) operators. The response parameters were growth performance, blood characteristics, lipid profiles, internal organ weights, carcass traits, gut morphology, intestinal microbiota, antioxidative biomarkers, immune responses and meat quality. Studies were used for the review if they reported the impact of spirulina on at least one of the response parameters of interest in broilers. Studies that reported the effect of spirulina on animals other than broilers were excluded. The retrieved studies were imported into Zotero (Version 7.0), and duplicates were excluded. A 2-step study selection approach was employed following the standard methods (Orzuna-Orzuna et al. 2022; Ogbuewu et al. 2025). First, the titles and abstracts of the identified studies were independently screened to exclude studies not on broilers and parameters of interest. Second, the full-text studies that met the title and abstract screening were used for the review. In total, 856 sudies were identified of which 95 studies that met the inclusion criteria were used for the review.

## Nutritional profiles

### Proximate composition

Results of proximate studies are routinely used in research to give a clue to the nutritional quality of feedstuffs. Table 1 shows that spirulina contains crude protein (CP), crude fiber (CF), ether extract (EE), carbohydrates, and ash. Liestianty et al. (2019) reported that the protein content in spirulina is about 62% of its dry weight (DW), which is higher than the 35% DW found in most high-protein source plant-based food. The use of spirulina as a supplement in human and animal food has been encouraged due to its high levels of proteins, ash, and ether extract, which according to Spinola et al. (2022) can be added to poultry feed in moderate levels to enhance broiler growth and productivity. The CP values (43.39–65.6%) and EE (0.61–16.46%) of spirulina as shown in Table 1 agrees with the values (50–70% and 5.6–16.6%, respectively) reported by others (Marrez et al. 2014). However, this CP value was slightly higher than that reported for soybean meal (SBM) and groundnut cake (GNC), which were 40–49% and 40–50%, respectively (Shehu et al. 2021). Proximate biochemical analysis of spirulina found that CP value of spirulina was comparable to the values of 43.3–48.50% reported for SBM (NRC 1994; Sauvant et al. 2004). Furthermore, the crude protein content of spirulina was similar to the value of 65.7% reported for fish meal by Aduku (1999). The high CP content in spirulina supports its use as protein supplements in animal and human food (Belay 2002). However, the presence of protein and pigment complexes linked to the thylakoid membrane in spirulina makes the hydrolysis of pirulina proteins hard (Böcker et al. 2020; Buecker et al. 2022). This necessitates the use of commercial enzyme blends to degrade the resistant spirulina cell wall components that limit nutrient bioaccessibility and bioavailability (Pestana et al. 2020; Spinola et al. 2024a).

**Table 1:** Proximate and mineral composition of spirulina

Items (%)	References								
	1	2	3	4	5	6	7	8	9
Crude protein	56.5	65.6	56.7-60.59	43.39	53.2	52.5	62.20	52.4	53.31
Ether extract	1.05	14.2	0.61*	16.46	1.90	6.56	6.92	0.63	9.25
Crude fibre	3.56	-	0.01–1.90	0.75	1.88		3.23	3.4.2	
Total sh	**-**	9.5	7.80–10.0	32.67	9.14	21.6	15.92	11.9	
Carbohydrates	**-**	18.7		16.97					23.38
Calcium	1.08			2.44	0.20	1.20	1.04*		0.27
Available P	0.93			6.27	0.10		1.92*		0.97
Copper					0.0002	0.095	0.002*		0.0009
Zinc					0.0025	0.010	0.0004*		0.002
Manganese					0.0162	0.010	0.007*		0.002
Iron					0.0164	12.95	0.034*		0.054
Magnesium	0.42					8.89	0.002*		0.32
Potassium						3.23	2.18*		1.70
Sulfur						0.01			

1 - Abdel-Moneim et al. (2022a, b); 2 - Khan et al. (2020); 3 - Park et al. (2018a)/Mullenix et al. (2022)*; 4 - Abd El-Hady and El-Ghalid (2018); 5 - Kolluri et al. (2022); 6 - Spinola et al. (2024a); 7 - Alwaleed et al. (2020); 8 - Sugiharto et al. (2018); 9 – Rahim et al. (2021); *Morsy et al. (2014)

The low CP value of 43.39% reported by Abd El-Hady and El-Ghalid (2018) for spirulina could be linked to the high salinity of the medium it was cultivated, as according to Ravelonandro et al. (2011), low CP content is linked to high salinity (Ravelonandro et al. 2011). The EE values of 0.63–1.90% reported by Mullenix et al. (2022) and Kolluri et al. (2022) were lower than the range of 14.2–16.46% reported by others (Khan et al. 2020; Abd El-Hady and El-Ghalid 2018). This observation may be attributed to the kind of solvent or extraction technique used (Pohndorf et al. 2016).

Proximate analysis results as displayed in Table 1 showed that the carbohydrate content of spirulina is within the range of 16.97 to 23.38% supporting the earlier reports that 15–20% DW of spirulina is carbohydrates (Chaiklahan et al. 2022). Polysaccharides, which serve as structural and storage components, make up the majority of the carbohydrates in spirulina (de Sousa eSilva et al. 2018). Spirulina contains carbohydrates such as rhamnose, galactose, xylose, glucose, and mannose (Spinola et al. 2024b), which bind with sulfur and calcium to enhance cell wall integrity (Hayashi et al. 1996). Additionally, spirulina has considerable levels of glycogen and other lower-molecular-weight carbohydrates, which serve as an energy reserve (Markou et al. 2012). Interestingly, spirulina has sulfated polysaccharides, such as sulfated rhamnoglucan heteropolysaccharide, calcium spirulina (Ca-SP), and α-pyranose, which have several pharmacological effects, including antiviral, immuno-modulation and anti-inflammation activities (Spinola et al. 2024b). This review also revealed that spirulina is high in ash (7.80 to 32.67%; Table 1), thus confirming the earlier reports that spirulina is an excellent source of minerals (Janda-Milczarek et al. 2023). These results support the reports of others (Rahim et al. 2021; Spinola et al. 2024a, b) that spirulina is high in minerals such as phosphorus, calcium, and magnesium required for cellular homeostasis and supporting metabolic processes. The current review shows that several studies were done on proximate analysis of spirulina (Table 1), with few studies targeting mineral quantifications. This review also revealed that spirulina is high in iron and potassium (Liestianty et al. 2019) when compared to maize and SBM, the main protein feedstuffs in chicken diets (Sauvant et al. 2004). There is evidence that minerals in spirulina have high bioavailability, indicating that they could be used as a feed supplement in chicken production. The observed variations in mineral values of spirulina as illustrated in Table 1 can be explained on the basis that the mineral content of spirulina varies with the mineral content of the growth medium, light intensity, cultivation condition, temperature, nutrient availability, and processing methods (Rahim et al. 2021).

### Amino acid composition

Several studies have shown that spirulina is high in both essential and non-essential amino acids (Table 2). Amino acids are organic compounds that are needed in various biological processes in the animal body. Research (Liestianty et al. 2019; AlFadhly et al. 2022) indicates that spirulina is an excellent source of essential amino acids (EAAs): 5.50 7.67% leucine, 1.00 -1.93% tryptophan, 1.40–2.39% methionine, 2.8–4.42% phenylalanine, 3.0-4.37% lysine, 3.3–4.88% threonine, 3.6% isoleucine and 4.5–6.37% valine. The high level of EAAs in spirulina implies that it can be used as a feed supplement in chicken feed to meet the daily needs of these EAAs. Table 2 shows that spirulina has excellent EAA profiles than SBM which is utilized as the major plant protein source in the livestock and poultry industries. The rich EAA in spirulina makes it a complete protein source similar to the values obtained in animal products, including meat and eggs (Wild et al. 2018). The excellent amino acid composition of spirulina (Evans et al. 2015) lends credence to its use as a food and feed supplement (Spinola et al. 2024b).

The role of dietary fiber in the movement of digesta and nutrient digestion is well known (Jha and Mishra 2021). Proximate studies (Table 1) show that spirulina is low in crude fiber compared to conventional fiber sources such as dried brewer’s grain (8.25%) (Yisa and Piental 2020). Rahim et al. (2021) showed that spirulina contains 30.38% neutral detergent fiber (NDF), acid detergent fiber (ADF), and 1.64% acid detergent lignin (ADL). The spirulina’s moderate NDF level shows that if consumed in excess, it can harm chicken performance. This moderate NDF value may be due to its rigid peptidoglycan, a semirigid, tight-knit.

**Table 2:** Amino acids profiles of spirulina

Items (%)	References					Mean	SBM^6^
	1	2	3	4	5		
Essential amino acids							
Histidine	0.9	1.38	0.78	0.93	1.0	0.80	1.26
Threonine	3.2	3.87	1.24	2.94	3.3	2.91	1.83
Methionine	1.2	4.20	1.24	1.49	1.4	1.91	0.66
Isoleucine	2.6	4.16	3.33	3.26	3.6	3.39	2.09
Phenylalanine	2.7	1.59	2.23	2.64	2.8	2.39	2.38
Leucine	5.2	1.47	2.79	5.07	5.5	4.01	3.58
Lysine	3.1	1.45	2.35	2.75	3.0	2.53	2.87
Valine	3.3	4.36	4.63	3.42	4.5	4.04	2.17
Tryptophan	-	-	-	-	1.0	1.00	
Non-essential amino acids							
Aspartate	6.2	3.05	4.79	-	6.0	5.01	-
Arginine	3.7	0.83	3.03	3.80	4.4	3.15	3.41
Cystine	-	1.88	0.51	-	0.7	1.03	-
Proline	2.4	3.05	2.36	-	2.7	2.63	2.38
Serine	3.3	1.20	2.47	-	3.3	2.57	2.09
Alanine	4.8	1.84	2.43	-	4.7	3.44	2.01
Tyrosine	2.4	9.06	4.19	-	3.0	4.66	1.75
Glutamate	8.6	1.58	2.38	-	9.2	5.44	8.26
Glycine	3.1	1.15	7.34	2.85	3.2	5.01	1.71

1 - Park et al. (2018a); 2 - Alwaleed et al. (2020); 3 - Abdel-Moneim et al. (2022b); 4 - Mullenix et al. (2022); 5 - Liestianty et al. (2019); 6 - Chen et al. (2010).

molecular complex found in its cell wall (Coelho et al. 2020), which can impair digestibility and nutrient uptake (Costa et al. 2023; Spínola et al. 2023). However, extrusion, heating, bead milling, microwave, freeze-drying, and sonication (ultrasonic cleaning), can significantly improve the digestibility of peptidoglycan thereby releasing the bound proteins (Buecker et al. 2022; Costa et al. 2023; Spínola et al. 2023)

### Vitamins composition

Table 3 summarizes the vitamin and fatty acid profiles of spirulina. This review shows that spirulina contains fat soluble vitamins [vitamin E (α-tocopherol) and vitamin A (β-carotene)], ascorbic acids (vitamin C), and B-complex vitamins, especially niacin, riboflavin, folic acid, and pantothenic acid, which are essential for cellular functions and nutrient (energy) metabolism (Park et al. 2018a; Rahim et al. 2021; Abdel-Moneim et al. 2022b; Kolluri et al. 2022; Spinola et al. 2024b). Studies have shown that spirulina contains vitamin B12, however, not much is known about its bioavailability and bioactivity (Liestianty et al. 2019; Janda-Milczare et al. 2023). Research also revealed that spirulina is a good source of chlorophyll and phycobiliproteins such as phycocyanins, allophycocyanins, and phycoerythrins (Aouir et al. 2017), which give spirulina its unique blue-green coloration (Christaki et al. 2011). The major pigment in spirulina is phycocyanin, a blue pigment-protein complex that makes up 47% of its dry weight (Bortolini et al. 2022). Rahim et al. (2021) reported higher phycobiliprotein content in spirulina than those obtained in earlier studies (Patel et al. 2005; Aouir et al. 2017). The reported difference in phycobiliprotein content in spirulina could be influenced by light intensity, temperature, and salinity (Rahim et al. 2021). Phycocyanin also acts as an accessory pigment in photosynthesis, helping chlorophyll to trap light energy, as well as its potent antioxidant and health properties (Christaki et al. 2011). The large amount of chlorophyll and carotenoids in spirulina helps it to grow in several aquatic environments (Park et al. 2018b). Furthermore, the carotenoids present in spirulina not only help in photosynthetic activity but also serve as antioxidants, neutralizing free radicals and preventing lipid peroxidation (Sousa et al. 2008).

### Fatty acids composition

Table 3 summarizes the fatty acids (FAs) composition of spirulina with capric acid and palmitic acid being the most abundant saturated FA (SFA). Spirulina is renowned for having ω-3 (omega-3), ω-6 (omega-6), SFA, unsaturated FAs (UFA), monounsaturated FA (MUFA), polyunsaturated FA (PUFA), which plays important functions in several metabolic and physiological processes in chicken (Spinola et al. 2022; Tavakoli et al. 2025). However, several variables can influence the concentration of FA, such as salinity, time of harvesting, and cultivation conditions (Tavernari et al. 2018). Also, α-Linolenic acid (GLA), γ-linoleic acid (ALA), capric acid, and palmitic acid are present in significant quantity in spirulina. Tavakoli et al. (2025) also found rich concentration of γ-linoleic acid (30.89%), palmitic acid (21.40%), linoleic acid (10.97%), hexadecatrienoic acid (9.90%), and γ-linolenic acid (8.82%) in spirulina. This observation agrees with Martins et al. (2021) and Spinola et al. (2022) who found that GLA, an ω-6 FA with anti-inflammatory effects, is the most abundant FA in spirulina. Similarly, spirulina contains 48.88% ω-3 (omega-3), 22.76% ω-6 (omega-6), 75.76% UFA, 26.06–71.63% PUFA, 23.65–63.43% SFA, and 8.98–9.40% MUFA, which is consistent with the earlier reports (Tavakoli et al. 2025). The high content of PUFA in spirulina indicates that it improves bioenergetics metabolism and maintains high-density lipoprotein cholesterol (HDL-C) concentrations (Li et al. 2018), meaning that spirulina can be added to broiler diets to produce lean meat.

**Table 3:** Vitamins and fatty acids composition of spirulina

Variables (%)	1	2	3	4	5
β-carotene (µg/kg)			294	982.4	169
α-Tocopherol (µg/kg)			29.5		1.15
Niacin					0.056
Riboflavin				0.24	0.12
Ascorbic acid					0.008
Folic acid					0.0008
Pantothenic acid					0.0018
Lauric acid	0.4				
Gadoleic acid	0.6				
Arachidic acid	0.4	8.93	0.19		
Ecosapentanoic acid		1.83			
Oleic acid	32.5	7.40	6.34	2.29	
Myristic acid	0.7	0.62	0.43	0.41	
α-Linolenic acid (omega-3)	21.4		15.8		
γ-Linoleic acid		26.93		14.06	
Linoleic acid (omega-6)	22.7	0.73	17.8		
Stearic acid	3.2	2.66	1.75	1.70	
Lignoceric acid	0.4				
Docosahexanoic acid		1.57			
Behenic acid	0.4	1.78	0.25		
Palmitic acid	15.5	43.06	39.7	24.64	
Palmitoleic acid	0.7	4.44	4.95	3.39	
Heptadecanoic acid			0.41	0.21	
Heptadecenoic acid			1.88		
Gondoic acid			0.91		
Caprylic acid				1.60	
Pentadecanoic acid				0.25	
Capric acid				34.62	
Tetradecanoic acid				0.58	
Vaccinic acid				3.14	
Eicosaenoic acid				0.24	
Hexadecatrienoic acid				0.25	
Gammalinoleic acid				11.51	

1 - Park et al. (2018a); 2 - Kolluri et al. (2022); 3 - Spinola et al. (2024a); 4 - Abdel-Moneim et al. (2022b); 5 – Rahim et al. (2021)

### Phytochemicals

Spirulina is rich in phytochemicals, a plant compound with varied structures and functions that exert potential health effects in animals and humans. Phenolic acids, polyphenols, steroids, alkaloids, saponins, terpenoids, and flavonoids are found in significant amounts in spirulina (Rahim et al. 2021) and may contribute to its several biological effects (Guldas et al. 2020; Bortolini et al. 2022). Phenolic acids account for one-third of the total phenol in spirulina with flavonoids making up the remaining amount. Rutin, naringenin, pyrogallol, vanillic, gallic, catechin, p-coumaric, hesperidin, quercitrin, and hesperetin were the main phenolic acids in spirulina (AlFadhly et al. 2022; Sinetova et al. 2024). Polyphenols such as ferulic acid and caffeic acid are found in large quantities in spirulina and are responsible for the antimicrobial, anticarcinogenic, antioxidant, antitumor, and anti-inflammatory activities of spirulina (Guldas et al. 2020; Ilieva et al. 2024). Spirulina has gained global attention in the pharmaceutical, feed, and food industries in recent times due to its several phytochemical bioactive substances, which elicit myriads of health benefits (Anvar and Nowruzi 2021; Bortolini et al. 2022). These beneficial effects are attributed to several secondary metabolites, polysaccharides, pigments, proteins, and fatty acids in spirulina (Bortolini et al. 2022). These pharmacological effects include antidiabetic, anticancer, antioxidant, antitumor, antiviral, anti-inflammatory, immunomodulation, cholesterol - and lipid-lowering properties (Anvar and Nowruzi 2021; Bortolini et al. 2022). The most widely investigated biological activities of spirulina are its antioxidant effects (Calella et al. 2022), which are attributed to its various pigments, especially carotenoids (lutein, β-carotene, and zeaxanthin), phycocyanin, and chlorophyll (Bortolini et al. 2022). Additionally, phycocyanin has been found to stop the synthesis of pro-inflammatory cytokines and decrease the activity of inflammatory enzymes like cyclooxygenase-2 (COX-2) (Fernández-Rojas et al. 2014). For detailed information on spirulina’s pharmacological properties, the recent review published by Spinola et al. (2024b) should be consulted.

### Mechanisms of action

The exact mechanisms by which spirulina enhances animal performance and well-being are not well-known. However, spirulina may performs its functions through a variety of mechanisms which in poultry includes: (1) Maintaining gut microbiota composition by encouraging the population of beneficial microbes and inhibiting the proliferation of pathogenic microbes through competitive exclusion and antagonism (Sugiharto et al. 2018; Shao et al. 2019; Alwaleed et al. 2020; Attia et al. 2023; Abdelfatah et al. 2024); (2) Having rich concentrations of potent antioxidant and anti-inflammation compounds (phycocyanin) that neutralize free radicals, activate cellular antioxidant enzymes, and downregulation of pathways leading to inflammation (Wu et al. 2016; Sahil et al. 2024; Spinola et al. 2024b); (3) Improving immune functions by suppressing inflammation and increasing production and activity of immune cells such as T lymphocytes, macrophages, and natural killer (NK) cells (Bortolini et al. 2022; Calella et al. 2022; Spinola et al. 2024b); (4) Altering metabolism by providing enzymes (amylase, protease, and lipase) that are vital for feed digestion (Harmantepe and Yılmaz 2025); (5) Improving feed intake and feed conversion efficiency by stimulating the absorptive capacity of the small intestine (Khan et al. 2020; Alwaleed et al. 2020; Attia et al. 2023); (6) Lipid and cholesterol lowering effects by producing phycocyanin and α-linolenic acid that regulate lipid metabolism (Spinola et al. 2024a); (7) Enhancing nutrient uptake and immune functions by acting as prebiotics (Harmantepe and Yılmaz 2025); and (8) Provision of nutrients, beneficial pigments, and bioactive compounds (Rahim et al. 2021; Kolluri et al. 2022; Spinola et al. 2024a).

### Growth performance

Several studies have demonstrated the growth-promoting effects of spirulina supplementation in broilers (Table 4). Khan et al. (2020) and Abdelfatah et al. (2024) found that spirulina supplementation at 0.1, 0.15, 0.2%, and 0.1, 0.3, 0.5%, respectively significantly increased feed intake and body weight gain (BWG) and reduced feed conversion ratio (FCR) in broilers. This outcome is in harmony with others (Zahroojian et al. 2013; Fathi 2018; Abd EL‑Dayem et al. 2021) who found improved FCR in chickens other than broilers offered spirulina at 1.5, 2.0, and 2.5%. The improved growth performance might be due to rich nutrient composition of spirulina, including excellent protein and EAA content required for muscle accretion (Rahim et al. 2021; Abdel-Moneim et al. 2022b; Spinola et al. 2024a). Spirulina contains bioactive compounds such as carotenoids, flavonoids, phycocyanin, and ascorbic acid with potent anti-inflammatory, antimicrobial, and antioxidant effects (Spinola et al. 2024b). One of the possible mechanisms of action of spirulina is the proliferation of growth of beneficial gut bacteria such as lactobacillus (Alwaleed et al. 2020; Attia et al. 2023; Abdelfatah et al. 2024). Better BWG in broilers fed spirulina-supplemented diet compared to those on control treatment as reported by Khan et al. (2020) could be ascribed to the ability of spirulina’s bioactive compounds to modulate mucosal immunity by assisting in the production of immunoglobulins favoring the multiplication of healthy gut bacteria (Shokri et al. 2014). This finding corroborates Kaoud (2015) who observed improved BWG in broilers on dietary spirulina supplementation. However, this is at variance with Shanmugapriya et al. (2015), who reported lower BWG in broilers fed 0.5 and 1.0% spirulina.

**Table 4:** Growth dynamics of broilers on spirulina supplementation

References	Dosage (%)	Feed intake (g/d)			BWG (g/d)			FCR		
		CTL	TMT	Diff (%)	CTL	TMT	Diff (%)	CTL	TMT	Diff (%)
Khan et al. (2020)	0, 0.1–0.2	81.28	84.28	3.69	46.01	49.86	8.37	1.93	1.84	-4.66
Abdelfatah et al. (2024)	0, 0.1–0.5	99.79	99.78	-0.01	60.8	62.7	3.13	1.64	1.59	-3.05
Shinde et al. (2018)	0, 0.04–0.06	101.77	106.07	+ 4.23	53.2	58.54	10.04	1.64	1.59	-3.05
Park et al. (2018a)	0,0.25-1.0	81.6	82.59	+ 1.21	41.17	44.47	8.016	1.91	1.81	-5.24
Abd El-Hady and El-Ghalid (2018)	0, 3.0–6.0	49.32	51.42	+ 4.26	49.32	51.42	4.26	1.88	1.78	-5.32
Kaoud (2015)	0, 0.1	119.43	119.27	-0.13	63.34	64.95	2.54	1.89	1.81	-4.23
Fathi et al. (2018)	0, 0.03–0.09	106.53	105.73	-0.75	56.94	60.41	6.09	1.87	1.75	-6.42
Pestana et al. (2020)	0, 15.0	124	121	-2.42	83.8	74.2	-11.46	1.48	1.63	10.14
Spinola et al. (2024a)	0, 15.0	222	158	-28.83	50.9	32.2	-36.74	1.61	1.78	10.56
Alwaleed et al. (2020)	0, 0.5–1.20	91.37	97.76	+ 6.99	62.43	75	20.13	1.46	1.31	-10.27
Sugiharto et al. (2018)	0, 1.0	80.94	80.31	-0.78	50.57	50.6	0.06	1.6	1.59	-0.63
Bonos et al. (2016)	0, 0.5-1.0				42.1	36.05	-14.37	2.07	2.12	2.42
Costa et al. (2024)	0, 15.0	248	254	+ 2.42	50.2	42.85	-14.64	1.64	1.9	15.85
Kharde et al. (2012)	0, 0.5–1.5	66.09	65.88	-0.32	22.4	25.95	15.85	0.34	0.31	-8.82
Swati et al. (2022)	0, 2.5-5.0				15.25	16.9	10.82	2.18	1.96	-10.09
Mullenix et al. (2022)	0, 10	6.515	6.275	-3.68	3.96	3.54	-10.61	1.63	1.75	7.36
Opoola et al. (2019)	0, 0.6–1.8	145.75	129.27	-11.31	52.4	57.5	9.73	2.78	2.2	-20.86
Ross and Dominy (1990)	0, 5.0–15.0				40.83	40.8	-0.07	1.78	1.78	0
Rubel et al. (2019)	0, 0.5–1.5	68.87	65.14	-5.42				1.45	1.29	-11.03
Abd El-Hady et al. (2022)	0, 3.0–6.0	117.46	119.38	_1.63	66	68.84	4.30	1.78	1.74	-2.25

*BWG* body weight gain; *FCR* feed conversion ratio; + = increase; - = Decrease; *CTL* control group; *TMT* treatment group; *Diff (%)* percentage increase

Kharde et al. (2012) and Shinde et al. (2018) reported that the inclusion of spirulina in the broiler diet reduced feed intake, which is contrary to the findings of other investigators (Khan et al. 2020; Abdelfatah et al. 2024). However, this supports the findings of Spinola et al. (2024a) that feeding 15% spirulina with and without commercial enzymes (pancreatin and lysozyme) to one-day-old male Ross 308 broilers for 35 days compared to the control treatment reduced feed intake and BWG by 29 and 38% and increased FCR by 10%. This corroborates the findings of Costa et al. (2024) on the adverse effect of supplementation of 15% spirulina with or without commercial enzymes on the growth performance of broilers. This implies that feeding 15% spirulina alone or in combination with commercial enzymes reduces growth performance metrics in broilers. Feeding a diet with up to 41% of SBM replaced by dried spirulina had no significant effect on growth metrics in broilers (Ross and Dominy 1990). A study by Evans et al. (2015) shows that at higher inclusion rates (> 10%), the gelation of the proteins is reported, which leads to reduced amino acid digestibility and increased digesta viscosity. In a similar study, Pestana et al. (2020) found a 2 to 11% reduction in feed intake and BWG, and a 10% increment in FCR in broilers fed a diet containing 15% spirulina with or without commercial enzymes. The poor performance in broilers fed 15% spirulina with or without commercial enzymes could be linked to increased digesta viscosity due to resistant cell walls of spirulina that limit bioaccessibility and bioavailability of nutrients in broilers by preventing the target substrates from having access to endogenous enzymes (Jonhson and Gee 1986). Overall, the variable growth performance data of broilers fed spirulina-based diets may be partly explained by chemical composition of spirulina, processing method, diet composition, and the level of spirulina in the diet (Bonos et al. 2016; Rahim et al. 2021). A previous study by Venkataraman et al. (1994) showed that the replacement of fish meal with up to 17% dried spirulina or GNC with up to 14% dried spirulina did not affect broiler performance. This confirmed the previous report that spirulina is a good source of digestible nutrients, including protein (Park et al. 2018a; Alwaleed et al. 2020; Khan et al. 2020), excellent amino acid profiles (Alwaleed et al. 2020; Abdel-Moneim et al. 2022b; Mullenix et al. 2022), and minerals (Alwaleed et al. 2020; Kolluri et al. 2022; Spinola et al. 2024a) and could be serve as feed supplement for humans and animals.

### Blood characteristics

The feeding of varying levels of spirulina to broilers increased red blood cell (RBC) count, hemoglobin (Hgb), and packed cell volume (PCV) (Jamil et al. 2015; Alwaleed et al. 2020). Likewise, Zhang et al. (2001) reported that spirulina increased the concentrations of RBC, white blood cells (WBC), and Hgb, whereas Fathi et al. (2018) revealed that spirulina supplementation at the levels 0.03, 0.05, 0.07, and 0.09% had no significant effects on Hgb, PCV, RBC, and red cell indices [mean corpuscular hemoglobin (MCH), mean corpuscular volume), and mean corpuscular hemoglobin concentration (MCHC)], monocytes and eosinophil counts in broilers. In a similar study, Zhang et al. (2001) found that spirulina increased the concentration of RBC, WBC, and Hgb in animals. Furthermore, Mariey et al. (2014) observed that dietary spirulina (0.3 mg/kg diet) increased WBC counts in broilers, while Fathi et al. (2018) found significantly increased WBC and lymphocyte counts and significantly reduced heterophils (H) / lymphocytes (L) ratio at 0.09% spirulina supplementation which could be due to the immunomodulatory effect of spirulina bioactive compounds (Khan et al. 2005). The improvement effect of spirulina on RBC may be partially related to its high concentrations of iron (90 mg/100 g) and B-complex vitamins, especially niacin, riboflavin, folic acid, and pantothenic acid, which help in the rapid formation of RBCs (Nasirian et al. 2017; Spinola et al. 2024b). Furthermore, spirulina contains antioxidant compounds that assist in protecting RBC and other blood cellular components from oxidative stress. In contrast, Sugiharto et al. (2018) and Alwaleed et al. (2020) indicated that spirulina reduced WBC counts and aspects of differential WBC. These findings were at variance with the findings of Jamil et al. (2015) and Lokapirnasari et al. (2016) that spirulina increased WBC levels in broilers. This observation may be ascribed to several factors such as the growth condition of spirulina, its chemical composition, the level added to the diet, and the age of broilers used for the study.

Blood chemistry parameters of broilers were influenced by dietary spirulina supplementation (Hajati and Zaghari 2019; Alwaleed et al. 2020). Blood proteins reflect the formation of proteins in the liver, which may be related to broilers’ growth performance and health status (Limdi and Hyde 2003). A study by Alwaleed et al. (2020) revealed that spirulina inclusion at 0.5 and 1.0% increased the concentrations of total protein in broilers while serum albumin concentrations were increased at 0.5% of spirulina supplementation. This suggests the high quality of protein in spirulina-based diets (Liestianty et al. 2019; AlFadhly et al. 2022). Fathi et al. (2018) noticed significantly increased serum globulin values in broilers offered 0.03 to 0.09% spirulina for 38 days but did not influence total protein and albumin levels. Pestana et al. (2020) reported that broilers fed 15% spirulina + 0.005% enzyme had significantly reduced serum uric acid levels, implying that spirulina improved nitrogen utilization in broilers, hence leading to reduced protein degradation (Metayer et al. 2008). Hepatic markers, including alanine aminotransferase (ALT), aspartate aminotransferase (AST), and alkaline phosphatase (ALP) are important indices used to assess liver function in farm animals (Ogbuewu et al. 2015). Serum ALT and AST levels were reduced at high supplementation levels of spirulina (0.07 and 0.09%) in broilers (Fathi et al. 2018), indicating that incorporation of spirulina to broiler diets did not compromise the normal function of the liver in broilers. This agrees with the results of Pestana et al. (2020).

The reduction effects of spirulina supplementation levels on lipid profiles, including total cholesterol (TC), triglycerides, low-density lipoprotein (LDL), high-density lipoprotein (HDL), and very low-density lipoprotein (VLDL) in broilers have been demonstrated (Zeweil et al. 2016; Abd El-Hady and El-Ghalid 2018; Alwaleed et al. 2020). The potential of spirulina to lower the lipid profiles of broilers that received spirulina-based diets at 0.5 and 1.0% (Alwaleed et al. 2020), 0.3 and 0.6% (Abd El-Hady and El-Ghalid 2018) and 0.05, 0.07, and 0.09% (Fathi et al. 2018) may reflect the ability of spirulina to inhibit the formation of cholesterol in the liver and its subsequent absorption from the gastrointestinal tract (GIT) of broilers (Mariey et al. 2012; Spinola et al. 2024b). The high level of PUFA in spirulina may help to reduce the serum lipid profiles of broilers (Hajati and Zaghari 2019). The hypolipidemic and hypocholesterolemic action of spirulina may also be related to the presence of phenolic compounds and C-phycocyanin, which inhibits the activity of fat-digesting enzymes in the chicken gut (Deng and Chow 2010).

### Immune functions

Khan et al. (2020) used 120-day-old male broiler chicks (Arbor Acres) which were randomly distributed into four groups of 30 broiler chicks each, with each group further allotted into three replicates of 10 broilers each. Group 1 had no spirulina (control 1), while Groups 2–4 received a basal diet supplemented with spirulina at 0.1, 0.15 and 0.2%, respectively. Results indicate that broilers on spirulina supplementation had better immune responses than the control. Based on the findings of Khan et al. (2020), spirulina supplementation at 0.1–0.2% supported immune functions in broilers. These findings are consistent with the work of Yusuf et al. (2016), who discovered that the replacement of subtherapeutic levels of antibiotic growth promoters with spirulina improved immune functions in broilers. Studies have shown that spirulina supplementation is an excellent source of important nutrients that may improve immune functions in broilers (Velten et al. 2018; Spinola et al. 2024b). Lysine, an EAA found in high levels in spirulina aid in the generation of cytokines and the proliferation of lymphocytes, resulting in improved immune systems in the face of ailments (Nasr et al. 2012). This inference was supported by Abdelfatah et al. (2024), who demonstrated that supplementation of spirulina to broiler rations at 0.1–0.5% improved the gut barrier function as shown by an increase in the expression of intestinal fatty acid-binding protein 2 (FABP2) that is charged with modulation of cell growth and proliferation.

### Antioxidative biomarkers

Pestana et al. (2020) noticed increased antioxidative enzyme capacity in broilers fed 15% spirulina with or without enzyme supplementation, which could be attributed to antioxidant components in spirulina, including sulfated polysaccharides, carotenoids, chlorophyll, phycocyanin, tocopherol, ascorbic acid, and phenolic acid (Kolluri et al. 2022; Spinola et al. 2024b). Research showed that broilers fed 0.1–0.5% spirulina exceeded the control treatment in terms of glutathione peroxidase (GPx), superoxide dismutase (SOD), total antioxidant capacity (TAC), and catalase levels. The GPx and SOD act as free radical scavengers within the cells (Ighodaro and Akinloye 2018). The inclusion of spirulina in the diets ofbirds has been discovered to alter their redox status; for instance, Park et al. (2018a) reported a progressive increase in GPx and SOD levels and decreased malondialdehyde (MDA) as spirulina levels increased from 0.25 to 1%. This observation was justified by the high content of antioxidant compound (C-phycocyanin) in spirulina (Spinola et al. 2024b), which is 16 times more effective than Trolox (a vitamin E analog) and 20 times more active than ascorbic acid (Romay and Gonzalez 2000). Numerous phenolic compounds (e.g., chlorogenic, salicylic, caffeic acids, trans-cinnamic, quinic, and synaptic) in spirulina may also add to its antioxidant activity, individually or mixed.

### Intestinal microbiota composition

In a study by Jeong and Kim (2014), it was observed that intestinal microbial ecology correlates with high productivity and efficient feed conversion in broilers. Research by Abdelfatah et al. (2024) indicated that spirulina (0.1, 0.3, and 0.5%) increased the cecal Lactobacillus population in a dose-dependent manner whereas the coliform population was increased at 0.3% and 0.5% supplementation levels. In a study with animals other than broilers, spirulina reduced ileal *E. coli* count but did not influence lactobacillus counts (Hajati et al. 2020). In contrast, others (Shanmugapriya et al. 2015; Fathi et al. 2018; Park et al. 2018a) found that spirulina increased the intestinal lactic acid bacteria (LAB) counts and decreased *E. coli* counts but had no effect on coliform count. The antibacterial effect of spirulina may partly be attributed to the activity of phenols and other bioactive compounds present in spirulina (Pradhan et al. 2014). In vitro studies revealed that spirulina extracts inhibit the proliferation of *Pseudomonas aeruginosa*, *Staphyloccocus aureus*, *Klebsiella pneumonia*, *E. coli*, Salmonella Typhi, *Candida albicans* and *E. coli*, (Kaushik and Chauhan 2008; El-Baz et al. 2013; Shao et al. 2019) while promoting the growth of LAB (Bhowmik et al. 2009).

Alwaleed et al. (2020) found that supplementation of 0.5 and 1.0% of spirulina in a broiler diet decreased the population of ileocecal *E. coli* compared to broilers without spirulina supplementation. This result supports the findings of Shanmugapriya et al. (2015), who reported a decreased *E. coli* count in broilers fed a diet having 1% spirulina. In a study other than broilers, Yusuf et al. (2016) found reduced ileocecal coliform count in quails fed with 2% spirulina. Reduced intestinal counts of *E. coli* or *S. aureus* have been demonstrated in broilers on dietary spirulina supplementation (Kaushik and Chauhan 2008; Nuhu et al. 2013). These antimicrobial effects could be attributed to its rich concentrations of C-phycocyanin, sulfonated polysaccharides, tocopherols, and fatty acids (El-Sheekh et al. 2014).

### Intestinal morphology

The gut acts as a selective barrier, absorbing nutrients and rejecting toxins and pathogens (Sharma et al. 2010; Pastorelli et al. 2013). Thus, maintaining a strong intestinal barrier is critical for the welfare of animals (Jeon et al. 2013). A weakened gut barrier encourages the passage of harmful substances, activating both the innate and adaptive immune systems (Senior et al. 2011). The beneficial effects of spirulina on gut morphology and absorptive capacity of villi in broilers have been demonstrated in the literature (Shanmugapriya et al. 2015; Yusuf et al. 2016; Khan et al. 2020). Spirulina is reported to enhance the proliferation of gut lactobacilli in broilers (Shanmugapriya et al. 2015) and quails (Yusuf et al. 2016). This finding agrees with Khan et al. (2020), who demonstrated improved gut morphology and absorptive capacity of villi in male Arbor Acres broilers fed a basal diet supplemented with 0.15 and 0.2% spirulina powder. The enhanced absorptive capacity of villi may be related to spirulina’s biological effects, which include antioxidant, immunomodulatory, and antibacterial activities. Based on the above findings, it is possible to conclude that spirulina, when included in the right quantity in broiler diets, might enhance the intestinal villi parameters, perhaps leading to improved BWG and FCR.

### Carcass traits, internal organs, and meat quality

The addition of spirulina at 0.15 and 0.2% to the diet of broilers increased the dressing percentage (DP) by 3.0 and 7.0% respectively (Khan et al. 2020). Similarly, Alwaleed et al. (2020) reported increased carcass traits in broilers fed 0.1% spirulina. This finding is consistent with the results of Kaoud (2015), who noticed that spirulina supplementation at 0.01% increased DP, liver, and gizzard weights in male Hubbard broilers, but had no significant influence on thymus and bursa weights. These suggest that spirulina-supplemented diets are superior to the control diet. It is evident from the findings of Abdelfatah et al. (2024) that the bursa, breast weights, and DP were higher in broilers fed 0.5% spirulina compared to those on 0, 0.1, and 0.3% spirulina supplementation. This is consistent with the results of Fathi et al. (2018) and Attia et al. (2023) that spirulina increased the weights of lymphoid organs in broilers exposed to heat stress conditions. The increased weight of lymphoid organs on spirulina supplementation suggests that spirulina improves immune functions in broilers. Khan et al. (2020) indicated that spirulina supplementation enhanced aspects of carcass traits and reduced abdominal fat (AF) content in broilers as documented by other investigatorss (Fathi et al. 2018; Moustafa et al. 2021). Similarly, Kaoud et al. (2015) found higher carcass yield, cut part weight (breast), and bursa weight in the spirulina treatment groups than in the control group. The improved carcass metrics (AF, DP, and breast muscle) can be related to spirulina’s excellent essential amino acid profile, which includes lysine and methionine. This shows that for enhanced DP and breast muscle weight, spirulina which is high in lysine, required for protein synthesis and muscle deposition, should be added to broiler rations in the right amount. In converse, Cheong et al. (2016) found no effects of incorporating about 8% spirulina in quail diets on the carcass and breast weights. To date, the exact reasons for the divergent results are not well known, but genetic differences, the composition of spirulina, and the quantity of spirulina added to the feed may explain the differences.

The color of raw meat is an essential quality attribute for the selection of meat by the consumer (Fletcher 1999). Chicken meat color is affected by nutrition and environmental variables (Venkataraman et al. 1994; Toyomizu et al. 2001). Spectrocolorimetric analyses indicated that supplementation of spirulina at a level between 4 and 8% in the diets of broilers aged from 21 to 37 days resulted in the orange-yellow color of meat compared to the control which was pale yellow (Toyomizu et al. 2001). The authors also observed that the orange-yellow color did not change as levels of dried spirulina increased from 4 to 8% in the feed. This agrees with the earlier reports by Venkataraman et al. (1994) that spirulina improved the color of thigh, skin, and breast muscles in broilers. The high carotenoid (β–carotene) and zeaxanthin pigments in spirulina could explain the improved orange-yellow color in broilers offered 4 and 8% spirulina (Spinola et al. 2024b). Another possible mechanism via which the meat color could increase in broilers offered spirulina may be related to increased myoglobin or iron levels in the meat due to the high levels of iron in spirulina (Spinola et al. 2024b). This implies that spirulina can be added to the broiler diet to enhance chicken meat color.

Based on the classification of the Food Advisory Committee (1990), thigh and breast muscles are recognized as lean meats because of their low-fat content, which is usually less than 5%. Pestana et al. (2020); Spinola et al. (2024a) discovered that total lipids in breast and thigh meats of broilers fed 15% spirulina with or without commercial enzymes satisfied the criterion of the Food Advisory Committee (1990). These findings are consistent with the values reported for thigh and breast meat by Ribeiro et al. (2014). Studies by Pestana et al. (2020); Spinola et al. (2024a) show that spirulina reduced the cholesterol content of thigh and breast meats in broilers. The authors also reported that the predominant SFA in the thigh and breast meat of broilers fed spirulina-based diets were palmitic and stearic acids. Palmitoleic and oleic acid were the predominant monounsaturated fatty acids, whereas linoleic acid and arachidonic acid were the most abundant PUFA. This aligns with reports of Tokusoglu and Unal (2003); Park et al. (2018a) that spirulina is rich in oleic acid, linoleic acid, palmitic acid, stearic acid, and γ-linoleic acid. The level of arachidonic acid was higher in breast muscles of broilers fed 15% spirulina with or without enzymes (Pestana et al. 2020), which might be attributed to the conversion of linoleic acid, which is the dietary precursor for arachidonic acid (Martins et al. 2021).

Minerals play an important part in several physiological processes in the animal’s body (Schaible 1941). A study by Spinola et al. (2024a) found that the addition of spirulina to the broiler ration had no effect on macro-mineral levels in breast meat, but did reduce the magnesium and potassium levels in thigh meat. The authors also noticed that 15% spirulina raised the sodium content and reduced the zinc level in both thigh and breast meat. The increased digesta viscosity in broilers fed 15% spirulina alone or with blends of commercial enzymes (Pestana et al. 2020) may impair the absorption of magnesium, potassium, and zinc from the gastrointestinal tracts.

### Economics of production

Khan et al. (2020) discovered that broilers fed 0% spirulina (control) had lower total costs than those fed 0.1–0.2% spirulina. The authors also found that groups fed 0.1–0.2% spirulina had better gross return per bird and net return than the control group. In a similar feeding trial, Abd El-Hady and El-Ghalid (2018) reported that spirulina supplementation at 0.3 and 0.6% reduced total feed cost by 10 and 6%, respectively compared to the control. In addition, relative economic efficiency (REE) increased by 19 and 27% in groups offered spirulina at 0.3 and 0.6%, respectively compared to the control group (Abd El-Hady and El-Ghalid 2018. Fathi et al. (2018) found higher total revenue, net revenue, and REE in broilers that received spirulina at 0.03 to 0.09% compared to the group without spirulina treatment. These findings confirmed the work of Kaoud (2015) and Mariey et al. (2014) who discovered that supplementation of up to 0.6% broiler feed resulted in increased economic efficiency in broiler production. Swati et al. (2022) revealed the addition of low levels of spirulina to broiler diets resulted in enhanced performance, however, it was observed that supplementation of spirulina at 5% alone or in combination with commercial enzymes is not economically feasible and can lead to a higher level of economic loss compared to the control group offered diet without spirulina and commercial enzymes.

## Conclusion and future research directions

This review shows that spirulina is an excellent source of nutrients, including proteins, vitamins (B-complex vitamins, tocopherols, ascorbic acids), pigments (phycocyanin, chlorophyll, and carotenoids), minerals (iron, calcium, phosphorus, and potassium) and PUFA. Additionally, spirulina is also a rich source of protein and EAA when compared to conventional vegetable protein such as soybean meal, the main protein source in broiler diets. Research also revealed that spirulina is high in chlorophyll, carotenoids, and phycocyanins, which give spirulina its unique blue-green coloration. The rich nutrient content and pigmentations of spirulina may be responsible for its several pharmacological properties, including antimicrobial, antioxidant, and anti-inflammatory effects.

This review demonstrates that the addition of 0.03–6.0% spirulina to the diets of broilers improved growth performance without adverse consequences on gut microbiota composition, gut morphology, blood characteristics, lipid profiles, antioxidative enzyme status, immune functions, internal organ, carcass traits, and meat quality. However, the introduction of high levels of spirulina (10–15%) alone or in combination with commercial enzymes into the diets of broilers reduced growth performance. The inclusion of high levels of spirulina in the diets of broilers increased digesta viscosity that tends to limit bioaccessibility and bioavailability of nutrients, which could explain the poor growth performance. The application of enzyme technology did not solve the problem of increased digesta viscosity, implying that a blend of other treatment methods such as enzyme and mechanical pre-treatments may be useful. In addition, the mechanisms underlying the poor growth performance of broilers fed high levels of spirulina with or without commercial enzymes is not clear and warrant further research. Further research is also required to ascertain the inclusion levels of spirulina that optimize broiler performance using a quadratic optimization model. There are also scanty published studies on immune functions, antioxidative biomarkers, intestinal morphology, and economics of production of broilers fed spirulina-supplemented diets, as a result, more research is needed in this direction.

This review revealed that supplementation of high levels of spirulina enhanced the breast and thigh meat color of broilers. Future research should focus on understanding the mechanisms involved in the meat color of broiler offered spirulina as it may yield new insights into practical applications for not only improving meat quality but also maintaining color uniformity in general meat or broiler meat marketing. This review showed that higher dietary spirulina levels reduced the content of magnesium and potassium in thigh meat while increasing the sodium and decreasing the zinc level in both thigh and breast meat. The reported decrease in the zinc level of both breast and thigh meat in broilers fed spirulina-based diets, poses a major concern for the nutritional quality of spirulina and calls for the enrichment of spirulina-based diets with zinc or cultivation of zinc-enriched spirulina. Furthermore, long-term trials should look into the potential health effects of eating broiler meat fed-spirulina diets, thereby providing a detailed understanding of its consequences for human health.

## Data Availability

Data will be made available on reasonable request.
